# A Systems Innovation Perspective on Implementation and Sustainment Barriers for Healthy Food Store Interventions: A Reflexive Monitoring in Action Study in Dutch Supermarkets

**DOI:** 10.34172/ijhpm.2024.8036

**Published:** 2024-05-12

**Authors:** Cédric N.H. Middel, Tjerk Jan Schuitmaker-Warnaar, Joreintje D. Mackenbach, Jacqueline E.W. Broerse

**Affiliations:** ^1^Athena Institute, Faculty of Science, Vrije Universiteit Amsterdam, Amsterdam, The Netherlands.; ^2^Department of Epidemiology and Data Science, Amsterdam University Medical Centers, Vrije Universiteit Amsterdam, Amsterdam, The Netherlands.; ^3^Upstream Team, www.upstreamteam.nl, Amsterdam University Medical Centers, Amsterdam, The Netherlands.; ^4^Amsterdam Public Health, Health Behaviors and Chronic Diseases, Amsterdam, The Netherlands.

**Keywords:** Food Store Intervention, Supermarkets, Netherlands, Monitoring, Implementation, Sustainment

## Abstract

**Background:** Healthy food store interventions (HFIs) are an important health-promotion tool, but face implementation and sustainment barriers. This paper aims to explore the underlying factors that produce these barriers using an innovative systems innovation perspective, through the case study of a multi-component HFI. The HFI was implemented in a minor, national, cooperative supermarket chain, in the Netherlands, a competitive market where price-based competition is the norm.

**Methods:** The HFI was implemented for 6-12 months, in six stores. It was implemented by the researchers, and maintained by store employees. The study applied a Reflexive Monitoring in Action (RMA) approach, meaning that the researchers monitored stores’ adherence to the HFI, via store visits, to identify potential issues. Subsequently, the researchers interviewed the store managers responsible for the intervention, to have them reflect upon the barriers leading to these adherence issues, underlying systemic factors, and potential solutions. The stores implemented these solutions, and during the next monitoring visit the researchers evaluated whether the barrier had been resolved.

**Results:** We found that the HFI often clashed with regular activities of the stores (eg, competing over the same spaces) and that store managers generally prioritized these regular activities. This prioritization was based on the greater commercial value of those regular activities (eg, selling unhealthy products) according to store managers, based on their beliefs and assumptions about commerce, health, and consumer preferences. Due to the limited resources of supermarkets (eg, people, time, space), and the HFI often not fitting within the existing structures of the stores as easily as traditional practices, store managers often neglected the HFI components in favor of regular store activities.

**Conclusion:** Our findings illustrate the systemic factors that produce implementation barriers for HFIs, and the dynamics by which this production occurs. These insights help future researchers to anticipate and respond to such barriers.

## Background

**Key Messages**

**Implications for policy makers**

- Health promotion in food stores is often obstructed by friction with the commercial goals of food retailers.
- Although the factors that drive food stores to prioritize commercial outcomes over health promotion are deeply embedded in food store organizations, there are actors in these systems that support change. These actors can be valuable allies for initiating and spreading health promotion interventions.
- Effective health-promotion interventions should (initially) be developed in a safe environment with limited interference from the system they seek to change, which also resembles real-world contexts.
- Such environments could be cultivated by stimulating the development of (alternative) food-retail formats in which health promotion is more institutionalized.
- This study adds to and expands upon existing evidence on implementation and sustainment barriers health interventions in food stores, and as such these implications can be generalized to food-retail outlets in general.

**Implications for the public**

Health-promotion interventions in food stores are often difficult to carry out in real-world settings due to implementation and sustainment barriers. These barriers are sometimes caused by factors inherent to the food store system. This study explored these factors through a systems perspective. We identified several important factors: Within food stores, commercial success is the primary goal, and the incentives within the organization (such as performance metrics) encourage commercial success and profit maximization. The dominant belief in the organization is that this goal is best pursued by selling unhealthy products, and therefore the processes and systems are optimized around promoting unhealthy products, which creates issues when the focus is shifted towards promoting healthy products. This tendency is exacerbated by limited organizational resources (time, people, space), which create pressure to prioritize the most commercially “valuable” activities. Through these insights, intervention implementation and sustainment, and therefore impact, can be increased, thus improving their contributions to public health.

Non-communicable diseases pose a major threat to global public health^1^ and unhealthy dietary behaviors are a major risk factor for these diseases.^2^ Modern food environments, particularly those in food retail (eg, grocery stores and supermarkets),^3^ are an important contributor to these behaviors because they offer a wide selection of unhealthy products.^4,5^ As such, “healthy food store interventions” (HFIs),^3^ which aim to promote healthier choices in food store environments through in-store strategies such as marketing mixes and choice architecture,^6^ have gained substantial interest in recent years.^7^

 However, these HFIs often encounter barriers such as consumer demands for unhealthy foods, supply and resource issues, or conflicting organizational values,^6,8^ which harm the implementation, sustainment, and scalability of these interventions.^9,10^ In 2022, an examination of reviews concluded that within implementation science there is a lack of research on factors that affect the sustainment of HFIs after their initial implementation.^6^ This review also called for the use of systems change approaches to improve HFI sustainment,^6^ aligning with another review, that illustrated how implementation and sustainment barriers can often be linked to systemic factors.^8^ Several recent studies have explored HFI sustainment,^11,12^ and have applied systems approaches prospectively.^13^ However, a case study following a systems approach is lacking.

 This paper addresses this gap through the case study of an HFI, following a systems innovation lens. Systems innovation focuses on identifying and changing the factors in a system which contribute to a complex problem (eg, unhealthy diets) through innovations such as HFIs.^14^ Through this lens, food stores can be seen as systems in which the promotion of unhealthy choices is inherent to how things are done in the pursuit of commercial success.^4,15^ This way of “doing” results from certain systemic factors, which enable and constrain what people in the system (eg, employees) can do.^16^ The goal of an HFI is to introduce new ways of “doing” (eg, selling different products) that promote healthier choices. The HFI is often not aligned with what the systemic factors promote, resulting in “systemic barriers.” Such barriers persist as long as the underlying systemic factors exist. Therefore, identifying and addressing these factors is vital for both initial implementation and long-term sustainment.^8^ More detail is provided under “Theoretical Framework” below.

 Based on this perspective, this study addresses the following question: “Which systemic factors produce implementation and sustainment barriers for HFIs, and how?” In doing so, this study provides a currently lacking systems innovation perspective on the implementation and sustainment factors of HFIs. For this purpose, a case study was performed of an HFI in Dutch supermarkets, which was initially planned and implemented by the researchers, and subsequently maintained by the supermarket organization, with the researcher only providing information.

 In systems innovation research, a common strategy for identifying and overcoming systemic barriers is Reflexive Monitoring in Action (RMA).^17^ This validated approach consists of continuously monitoring and adapting what is implemented, to address encountered barriers and improve implementation and sustainment. This study provides a case example of the application of RMA for an HFI. More information is provided under “Study Design.”

## Methods

 The following sections cover the theoretical framework, context, intervention, study design, participants, data collection, researcher characteristics, data processing and analysis, and validity.

###  Theoretical Framework

 This study takes a systems innovation perspective on HFI implementation and sustainment. A previously developed framework was used,^8^ which integrates and expands the Consolidated Framework for Implementation Research^18^ with systems innovation theory, through the constellation perspective.^16^

 This framework defines individual food stores and their organizations (eg, supermarket chains) as systems ie, networks of actors and factors that interact to achieve a goal.^8^ The goal of these systems would be to make a profit by selling food to consumers. Individual food store systems are “subsystems” of the organization to which they belong (“food store organization system”). This means they are a part of the greater whole, but also have clear boundaries separating them from other parts of the organization (eg, other stores, central office).^14,16^ Sub-systems are often influenced by factors in their overarching system (eg, top-down decisions from an organizational director).

 In the constellation perspective, each system is a constellation of cultures, structures, and practices.^16^ The *culture* (values, beliefs) and *structures *(rules, boundaries, resources) of a constellation guide *actors* (people who work within a system) to carry out specific *practices *(routine activities), which transform resources into goods and services (eg, using human capital to sell food).^16^ Carrying out practices also produces legitimacy and meaning for the cultures and structures in the system. This creates a self-reinforcing loop in which the influence of systemic factors that stimulate actors to perform a certain practice, is strengthened by performing that practice.^16^

**Figure 1:**
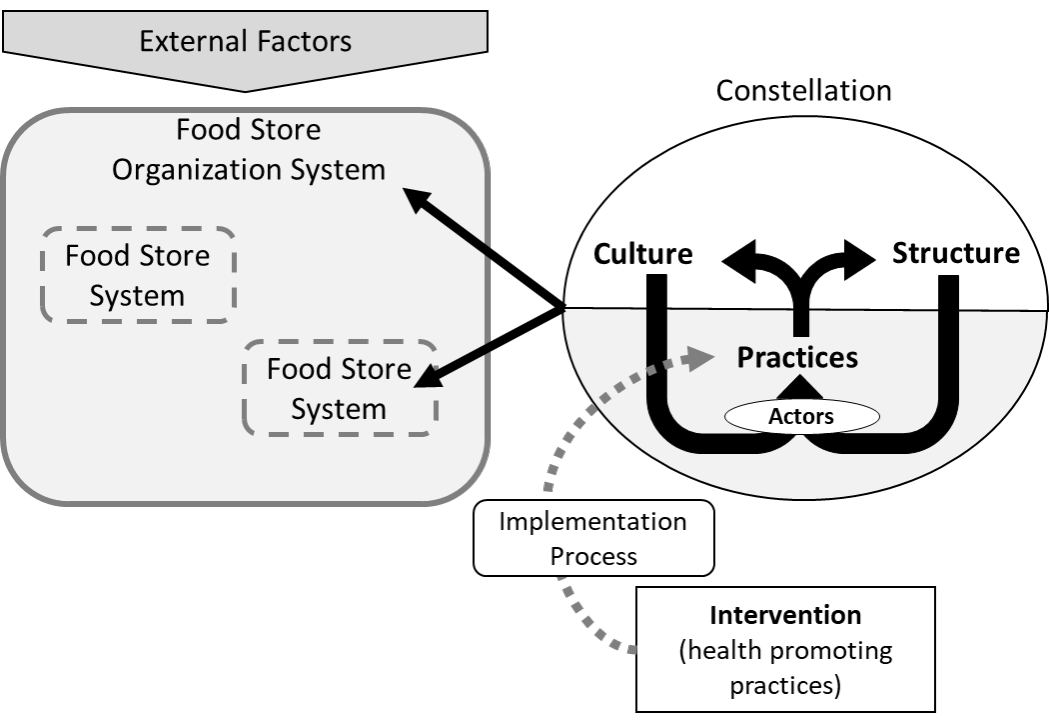
The Theoretical Framework for Healthy Food Store Implementation. Individual stores and the overarching organization are systems, with stores being sub-systems of the overarching system (indicated by dotted borders). These systems are constellations of cultures, structures, and practices. Interventions represent a novel category of health-promoting practices. The implementation of these interventions into existing systems can be influenced by pre-existing cultures and structures, the nature of the intervention itself, the implementation process, and external factors.

 In this framework, *interventions* (eg, HFIs) are conceptualized as new practices, that are explicitly different from the existing practices in a certain system. Implementation is defined as the integration of these new practices into an existing system (in this case the stores).^18,19^ The *implementation process* by which this integration is performed can affect the success of this implementation.^8,18^ For the complete framework, see Figure 1.

 Because interventions deviate from what the pre-existing cultures and structures (the systemic factors) promote, these factors will often constrain actors in performing the new intervention-related practices, which is what we define as systemic barriers.^19^ Such barriers can occasionally be overcome during initial implementation (eg, by allocating additional resources). However, over time the barriers will be reproduced by the underlying systemic factors. This reduces intervention sustainment unless these factors are directly addressed.^19^ The implementation and sustainment of the HFI can also be affected by *external influences*, such as outside the store and organization systems (eg, social, political, economic, natural factors, and events).^16,18^

###  Context

 This study was conducted in the Netherlands, where supermarkets are the primary food retailer.^20^ The Dutch food-retail market is highly competitive and saturated. It mainly competes on prices, although marketing campaigns framed as health promotion (eg, discounts on vegetables) are increasingly common.^21,22^ The researchers collaborated with supermarket chain Coop, a consumer cooperative,^23^ which primarily operates in the North, East, and South of the country, with a 3.9% market share (314 stores) in 2020.^20^ Coop’s customer panels have recently tasked the organization to focus on social issues, including health promotion.^24^

 This study was embedded within a randomized controlled trial (RCT).^25,26^ The RCT included six intervention (coded A-F) and six control stores, located in the South and East of the Netherlands. Stores were randomly selected from all stores suitable for the RCT (no near competitors, in areas with low socioeconomic status).^25^ As such, these stores were located in areas that were more remote and rural than average in the Netherlands, but otherwise representative. The control stores were not part of this study, and therefore not discussed further. We henceforward refer to the intervention stores as “stores.” More information on the RCT is reported elsewhere.^25,26^

###  Intervention

 The HFI examined in this study was co-created with actors from the Coop organization to shift sales from unhealthy towards healthy products in a commercially and practically sustainable way. Details on this process are reported elsewhere.^19^ The HFI consisted of fifteen components, which adjusted the presentation of products (eg, facings, placement), introduced signage (eg, posters, tags), or adjusted the prices of products (Table 1). Some components would remain the same for the entire 6 or 12 months, whereas others would change after a defined period (Table 1). Each store would implement all components.

**Table 1:** The Components of the HFI, Their Descriptions, and the Frequency of Component Adjustments to Promote Different Products

**HFI Components**		**Description**	**Adjustment Frequency**
Product presentation	Check-out presentation	A check-out with only healthy products	Never
	Shelf positions	Healthy products placed at more visible positions on the shelves	Never
	Head-shelf presentation	A head-shelf presentation with only healthy products	Every month
	Basket presentations	Baskets presented along the aisles, which present healthy products	Every month
Signage	Shelf tags	Small tags, next to price tags on the shelves, indicating a desirable characteristic for a specific (healthy) product	Never
	Posters	Small posters near healthy products, indicating a desirable characteristic for a group of products	Never
	Feedback strip	Feedback strip underneath healthy products, providing positive reinforcement to customers choosing the product	Never
	Banners	Banners hanging from shelves near healthy products show images of various healthy products to inspire customers	Never
	Check-out divider bars	Signage on divider bars at the check-out explains the themes (“tasty,” “popular,” and “quick”) used on signage, and encourage healthy choices	Never
	Shopping basket placemats	Placemats in shopping baskets explain the signage themes (“tasty,” “popular,” and “quick”) and encourage healthy choices	Never
	Cart boards	Boards on front of shopping carts, explain the signage themes (“tasty,” “popular,” and “quick”) used on signage, and encourage healthy choices	Never
	Cart handles	Stickers on handles of shopping carts, explain the signage themes (“tasty,” “popular,” and “quick”) used on signage, and encourage healthy choices	Never
	Shelf cards	Large, monthly changing shelf cards to draw additional attention to a specific product, follow same signage themes associated with desirable product characteristics as shelf tags	Every month
	Price cards	Cards indicating healthy products, which have been reduced in price as part of price change components, follows same signage themes associated with desirable product characteristics as shelf tags	Every month
Pricing	Price mutations	Upward (tax) and downward (subsidy) adjustments to product prices	Every month

Abbreviation: HFI, Healthy food store intervention.

 Throughout 2020, the researchers met with store managers and relevant actors in the central organization to discuss the HFI and its implementation. The researchers would assist with the initial placement of signage in each store, but subsequent maintenance and adjustment of the HFI was the responsibility of the stores. The HFI components were embedded in the organizational processes and systems for signage, product presentation, and price management where possible. A shared folder was set up with documents specifying the correct places for signage, and products approved for certain presentation spaces. Training sessions for store employees were planned, but due to the COVID-19 pandemic, they could not take place. Instead, store managers were asked to inform the relevant employees individually, supported by informative documents drafted by the researchers. The HFI was implemented in 2021. Four intervention stores (A-D) implemented the HFI for 12 months (April 2021–April 2022), and two for six months (November 2021–May 2022). This study followed all stores for their full participation period.

###  Study Design

 This study followed an RMA approach,* a validated qualitative approach for analyzing and overcoming *systemically embedded barriers for implementing interventions.^17^ RMA follows a cyclical pattern, consisting of: (1) *monitoring*: observe which parts of the intervention encounter issues (eg, low adherence), (2) *reflection*: reflect with the people implementing the intervention on what barrier causes these issues, what underlying factors are responsible, and how this can be resolved, and (3) *action*: the proposed solutions to the encountered barriers are implemented. Subsequent cycles monitor for and reflect upon new barriers, but also the success of the solutions put in “action” (and if needed, explore alternatives). By going through multiple such cycles “low-hanging fruit” barriers are resolved, while barriers that are caused by systemic factors will remain. These remaining barriers are explored in more depth through additional reflection steps until the responsible factors are identified and resolved.^17^

**Figure 2:**
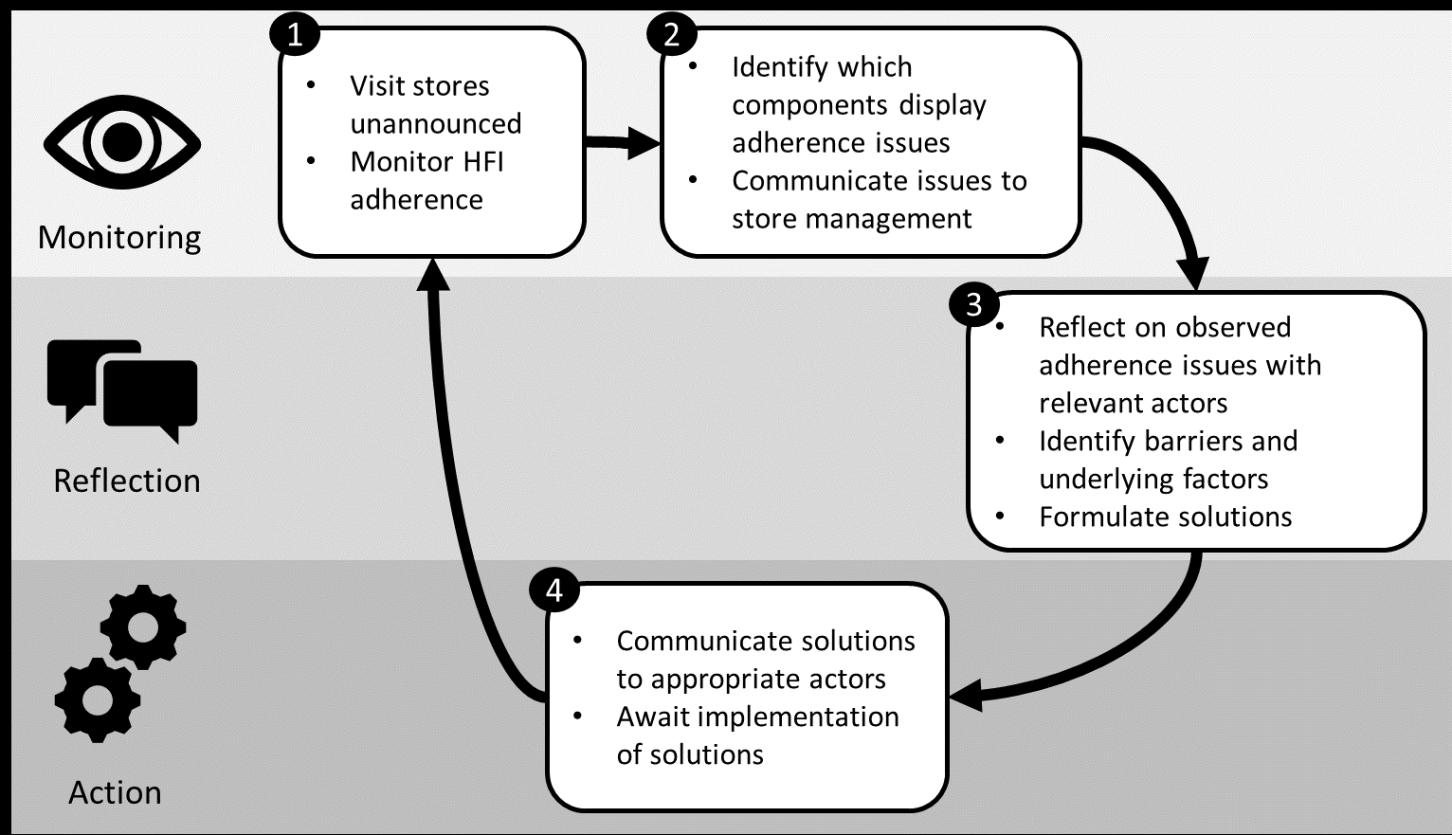
Study Design Organized Along the Main Steps of Reflexive Monitoring in Action. Abbreviation: HFI, healthy food store intervention.

 In this study, RMA was applied as follows (Figure 2):

####  Monitoring

 During the entire study, regular unannounced monitoring visits were conducted to each store. On these visits, researchers observed how closely the implementation of the HFI followed the planning. The observations of each visit were recorded on a checklist. A summary of observed adherence issues was shared with the store manager, as a feedback and validation strategy.

 The checklist (Supplementary file 1) was developed by CNHM and the researchers involved in the RCT. For each HFI component (See Table 1), the checklist listed the appropriate options out of the following characteristics: (1) “is the correct product promoted?” (2) “is it in the correct position?” (3) “are prices adjusted correctly?” and (4) “is it undamaged, clean, and readable?” Each characteristic was individually scored on a 5-point Likert scale, with 1 indicating approximately 0%-20% adherence, and 5 representing 80%-100% adherence (the acceptable range). The purpose of these scores was to inform the reflection interviews (discussed below), and thus the wide range in adherence covered per point was deemed acceptable.

 The visits were conducted individually, primarily by CNHM. Research assistants conducted multiple visits in April–May 2021 and July 2021 due to time constraints. These research assistants received training on how to conduct the observations and reviewed their scoring with CNHM afterward to ensure consistency. The first stores (A-D) were visited every other week to facilitate the implementation process. Subsequently, visits became monthly for up to six months and bi-monthly after six months (Table 2).

**Table 2:** Time Intervals for Monitoring Store Visits to Assess Healthy Food Store Intervention Implementation

**Stores** **Implementation Period**	**Frequency** **Month 1-2**	**Frequency** **Month 3-6**	**Frequency** **Month 6-12**
A, B April 2021–March 2022	Every 2nd week	Every month	Every 2nd month
C, D May 2021–April 2022	Every 2nd week	Every month	Every 2nd month
E, F November 2021–May 2022	Every month	Every month	-

####  Reflection

 Following each monitoring visit, CNHM set up an interview with the store managers or other store employees responsible for the intervention implementation. In these interviews, the interviewer and participants discussed observed adherence issues and reflected on responsible barriers, underlying causes, and how these issues could be resolved. Every second month, CNHM also set up a group interview with central office actors that were involved in the implementation process (eg, price management, shelf planning). These combined interviews were the primary data collection method.

####  Action

 The solutions that came out of the interviews were communicated via email or telephone to the relevant actors in the Coop organization by CNHM if these people were directly involved in the study or through a liaison within the organization if they were not. Solutions which were potentially beneficial to other stores were also communicated to those managers. Once communicated in this manner, implementing the solution was the responsibility of these actors and its effectiveness in addressing the associated issue would be observed during the next observation visit.

###  Participants

 There were 18 participants in total. Twelve of these were actors responsible for implementing the HFI in the intervention stores (A-F), including (assistant)managers (n = 11), and employees appointed to oversee components (n = 1). The remaining six were actors in the Coop central office involved with the organization of these components, who were responsible within the organization for corporate social responsibility (n = 1), marketing (n = 2), space planning (n = 2), and general cooperation matters (n = 1).

###  Data Collection

 As noted previously, data was collected via interviews (individually and in groups) which were held by video or phone call (following COVID-19 restrictions). The interviews were conducted by CNHM and a research assistant in April-May 2021. Interviews were recorded with participants’ consent (See ethics). Store managers and store employees were interviewed after each monitoring visit to their store, individually or together if responsibility for the HFI was shared. Central office actors were interviewed every second month, in groups.

 The interviews were semi-structured, based on the principle of a Dynamic Learning Agenda,^27^ which is commonly used in RMA. Practically, this meant the following: during each interview, any barriers, their underlying causes, and proposed solutions, were noted on a list (the “agenda”). A separate list was maintained for each store. Central office actors had a separate agenda with organization-wide barriers.

 Each interview started with a brief discussion of each point on this agenda for the appropriate store, to see if the barrier still existed or was resolved. This was informed by observations made during the preceding monitoring visit. If resolved, the barrier was removed from the agenda. If not, the researcher and interviewee(s) reflected on why the attempted solution had not worked and if underlying issues had been missed, and attempted to formulate a new solution.

 In the second part of the interview, the interviewer and participant discussed any adherence issues observed during the monitoring visits that had not been discussed previously in relation to the barriers already on the agenda. For each issue, the researcher and interviewee(s) reflected upon the barrier responsible for the adherence issues, underlying causes, and add these to the agenda. Finally, solutions were explored to resolve these new barriers.

 If interviewees were unable to formulate a solution for a barrier, it would be discussed with other participants in following interviews to gather additional input and perspectives. With this additional input, new attempts were made to formulate a solution, in the next interview. At the end of the interview, the interviewee(s) could discuss other topics and questions.

 Occasionally, there were spontaneous talks with store actors (management, employees) during monitoring visits, in which relevant information was discussed. These were recorded via fieldnotes.

###  Role of the Researchers

 The research activities were coordinated and primarily performed by CNHM. Leading up to the study, CNHM had contact with the actors at the stores involved in the intervention to explain the study, answer questions, and manage expectations regarding the role of CNHM (to observe and explore problems). These efforts facilitated mutual trust and understanding, which led to the participants being open in discussing implementation barriers, wider organizational issues, and potential solutions.

###  Data Processing and Analysis

 The audio recordings of the interviews were transcribed verbatim, or in case of short interviews with low information density, summarized. Transcripts, summaries, and field notes were analyzed through a qualitative content analysis following a combined deductive and inductive approach with semi-open coding.^28^ Initial codes were based on the theoretical framework (external factors, organizational and store culture, structure, and practice, implementation process, intervention), and the typology of the HFI components (See Table 1). The unit of analysis was factors that produce implementation and sustainment barriers.

 Coding was done by CNHM using Atlas.ti software.^29^ First, all documents were read, and every passage which discussed a barrier for the HFI, underlying factors, or solution to these factors, was noted. These passages were coded with (1) summarizing codes for factors that produce barriers, and attempted solutions, and (2) codes noting the involved components. Codes were re-used for passages discussing the same subjects. Interactions between factors were registered as links between their codes in a code network. Codes summarizing factors were categorized under the concepts of the theoretical framework. Codes summarizing solutions were linked to their associated barriers. Where appropriate, codes summarizing related concepts were placed under a new overarching code.

 To synthesize our results, an overview was developed, in which all codes representing factors were listed under their primary associated domain of the theoretical framework. For each factor, we drafted a brief description, summarizing the content of the passages associated with the code, in the table. We included references to the links of each factor with other factors (meaning there were interactions between them) and HFI components (meaning it affected these). Based on this table, a comprehensive narrative was drafted. These synthesized results were shared with our primary contact person in the Coop organization, for dissemination.

###  Validity

 Monitoring visits were primarily conducted by CNHM, and occasionally by assistants (April-May 2021, July 2021). To ensure internal validity, CNHM discussed the components and the proper use of the checklist with the research assistants in advance. CNHM reviewed the completed checklists and discussed noteworthy or unclear scores/comments with the researcher who completed them. Summaries of the observations were sent to the store managers as a validity check.

 Due to the interview structure, barriers that were not immediately resolved would be discussed multiple times with the same participant, which reduced the risk of misunderstandings regarding such a barrier between the researcher and interviewee. An early draft of the Results section was shared with our primary contact in the Coop organization, for validation. Furthermore, several of the barriers were encountered in multiple stores, providing multiple complementary perspectives on the situation. These factors increased the internal validity of the interviews. Finally, CNHM and a research assistant double coded the interviews conducted in the first two months of the study, and further coding was based on the resulting consensus.

## Results

 This study explored the question “which systemic factors produce implementation and sustainment barriers for HFIs, and how?” For this purpose, the adherence of an HFI was monitored, and observed issues were reflected upon to identify underlying factors.

###  Monitoring Outcomes

 An overview of the adherence of all HFI components is presented per store in Supplementary file 2. Components that were planned to remain unchanged for the entire study period generally displayed higher adherence, except for the healthy check-out presentations, which showed low adherence. Various components showed lower adherence in the initial weeks, which generally increased afterwards.

###  Implementation and Sustainment Factors

 Several systemic factors were identified as contributors to implementation and sustainment barriers. These were exacerbated by the factors related to the intervention, implementation process, and external factors. An overview of all factors, the affected HFI components and applied solutions is provided in Supplementary file 3. Below we first discuss factors related to the intervention, followed by the implementation process, the major systemic factors, and finally external factors.

###  Intervention

 Two factors related to the HFI played a major role in the observed systemic barriers: First, the *products* that certain HFI components promoted differed substantially from the products that stores usually promote. As such, store employees often had limited experience with promoting these products, and little trust in their commercial value. As a solution, additional information on these products (eg, profit margin) was provided and the product selection was adjusted based on feedback from store managers. Second, the *workload* of certain HFI components was substantial. As a solution, some components were reduced in size and scope, and further integrated in automated processes where possible.

###  Implementation Process

 The implementation process suffered from several shortcomings: First, the store managers and employees received limited* information and training* beforehand about the HFI, such as how it could affect their usual work, and how it would interact with performance metrics. To address this, the researchers informed store managers throughout the study, and provided educational materials in breakrooms for store employees.

 Second, *communication issues* existed between the researchers, central office and stores: These issues included researchers not being informed when store managers changed, information on HFI components being unclear or difficult to find for stores, stores not indicating to the central office that they needed more work hours, failure to set up meetings in which stores could exchange experiences, and limited feedback towards the stores regarding the preliminary outcomes of the HFI (due to issues with receiving the necessary data from the organization). Several solutions were implemented: sending information and reminders via email (the main communication channel for stores), simplifying how information on the HFI was presented to stores, researchers requesting additional work hours for stores, and developing an informative flyer with preliminary outcomes of the HFI.

 Third, *organizational support* for the HFI was often perceived as lacking in the stores. Store managers noted that their employees lacked initiative and diligence in implementing HFI components. The managers blamed lack of interest and understanding about the project and health promotion efforts. This was likely due to the lack of training provided to employees in advance. Consequently, store managers shouldered most of the HFI-related workload. As a solution, educational materials aimed at store employees were placed in store breakrooms. Additionally, store managers felt they were limited in their ability to effectively carry out the HFI due to restrictive performance metrics and activities (eg, campaigns, reorganizations), which reduced their motivation. These metrics and activities could not be addressed within the project’s scope. To mitigate the issue, the organizational management sent a letter of appreciation and encouragement to the store managers.

 Fourth was *integration* issues: There were notable timing differences for product presentations and signage between the HFI and how stores usually operated. Reminders were sent to stores to remind them of these schedules. In addition, the integration of presentation components’ “space planning” and “replenishment” IT systems was initially lacking. Furthermore, for certain targeted products, the optimal stock range was initially uncertain, necessitating trial and error to determine.

 Fifth was the *supply* of intervention materials. Stores had to order materials for signage themselves, which they often forgot to do, or materials were unavailable. The result was that these components were poorly maintained. As a solution, researchers frequently checked whether stores had all the necessary materials, and reminded them where and how to order these.

###  Store and Organization System

 The following sections will describe the barriers encountered in practice, followed by the underlying systemic factors relating to structure and culture.

####  Practice

 First, *friction* between HFI components and regular store practices was a recurring barrier. This friction was often a result of the HFI and practices competing over limited resources (work hours, space) and organizationally mandated activities that conflicted with the HFI. For example, an organization-wide program demanded store managers to assess and improve the commercial viability of their presentation spaces. Furthermore, organization-wide price promotions sometimes clashed with the pricing component. In such cases, store managers felt pressured by their superiors and performance metrics to prioritize the (perceived as more profitable) organizational activities and regular store practices. As a result, the HFI components usually seemed to have low priority in the stores.

 This *prioritization* led to stores not maintaining HFI components that cost substantial resources (time, people, space). This were generally the components with significant friction, substantial workload, unclear information, or lacking materials. The researchers attempted to reduce friction by lobbying for additional resources at the central office, and focusing the intervention on products perceived as profitable, with limited success. Store managers were regularly reminded to implement neglected components. Central management sent messages to stores to stress the importance of HFI and express organizational support. As a last resort, the researchers asked store managers to prioritize specific components that were expected to have the greatest impact on promoting healthier diets.

 Second, several *events* posed barriers to the HFI. A reorganization led to store employees gaining new responsibilities and additional training. Furthermore, the managers of several stores were replaced during the study. This added workload for the employees and led to general and HFI-specific knowledge being lost. In the final month of the study, the organization decided to merge with another chain, and in several stores the preparations for refurbishing led to the HFI being abandoned prematurely.

 Third, customers sometimes posed challenges for the HFI. They expressed discontent about specific components, particularly when incorrect prices were displayed due early issues with the price component. Customers also occasionally (unintentionally) removed or damaged signage. These issues were mainly resolved by addressing issues with the price component and attaching signage more securely.

####  Structure

 Several of the barriers observed in practice were a result of several structural factors which did not align with the HFI:

 First, *human resources*: Stores often experienced shortages in both people and time. As a result, they struggled to fulfil their regular tasks, let alone those related to the HFI. This mostly impacted the components that required frequent adjustments, as they demanded more attention. Additionally, there was substantial turnover among the study’s key collaborators. Store managers often changed during the study, and key personnel at the central office became increasingly unavailable in the final months due to the upcoming merger. This resulted in the loss of crucial knowledge at all levels, and previously resolved issues reappearing. Although employee shortages could not be solved, the turnover of key actors was managed by immediately contacting their replacements to inform them.

 Second, *knowledge resources*: Store managers and employees often lacked knowledge of healthy products (eg, profit margins, improved sales following promotions). This made it difficult to determine which healthy products were profitable. Some of this information was available within the organization and was planned to be shared with the stores going forward. Store managers and employees also rarely knew which products were healthy, and therefore could not always recognize when a wrong product was promoted. The organizational product database did not initially include this information either. When it was eventually added, by linking it to a database from the Dutch Nutrition Centre, it was still rarely used.

 Third, *available space*: Due to the limited space for presentations in stores, the HFI would often occupy space otherwise used for regular promotions, for, (what were perceived as) less profitable and popular products. This factor led to substantial friction as discussed above between presentation components and regular store practices. Stores were offered additional presentation spaces (eg, baskets) by the organization, but due to the lack of floor space, none pursued this.

 Fourth, *products*: Store actors frequently disagreed with the central office’s product choices for presentation, citing factors such as shelf life, size, price, profit margins, dietary function, and popularity as reasons for the products being commercially unsuitable for the designated location. As a solution, store actors were given the freedom to order products they approved, from a list of healthy options. However, they often forgot this, or complained that the profit margins of these products were not listed. This data was eventually added. Moreover, stores occasionally overstocked certain products, risking expiration. In these cases, store managers prioritized promoting these products in spaces meant for healthy products. A solution to this problem was not found.

 Fifth, *processes and systems*: Throughout the trial, the IT system for prices created multiple barriers. Initially, it could not differentiate between the HFI-related and regular price changes, causing issues with placing the HFI price cards. The solution was to schedule HFI-related changes on a different day, but this led to employees forgetting about them. Additionally, software bugs occasionally resulted in incorrect store prices, leading to discontent customers. Transitioning to a new system, a plan preceding the study, resolved these issues but introduced new ones, leading to incorrect prices and frustrated customers and store managers. This was later discovered to be an organization-wide problem unrelated to the HFI. Nevertheless it harmed the perception of the HFI.

 Sixth, *performance metrics*: The organization evaluated store performance through metrics focusing on quantitative commercial factors such as hours worked, salary costs, waste, and turnover. These evaluations also determined store managers’ bonuses. Such metrics therefore significantly influenced store priorities and resource allocation. Store managers also noted an organization-wide push to improve on these metrics, especially hours and salaries, through reorganizations and programs. To promote prioritizing the HFI, the central office assured store managers that the metrics would be adjusted to accommodate the HFI’s impact. However, as mentioned earlier, store managers still felt pressure to optimize their performance against these metrics and allocated resources accordingly.

####  Culture

 Furthermore, two cultural factors also contributed to the barriers for the HFI observed in practice:

 The first factor was *beliefs* that did not align with the HFI: Several store managers perceived some HFI components as harmful to their aforementioned performance metrics. They worried this could negatively affect their personal evaluations and performance bonuses. Despite efforts from the central office to clarify that the HFI would not negatively affect these evaluations or bonuses, these concerns persisted and several stores prioritized more “commercially valuable” activities.

 Furthermore, store managers often disagreed with the healthy products selected for presentation components, as they believed these would not perform in such spaces. As a result, they occasionally deviated from or completely ignored, these components. As a solution, these managers were asked to advise on the product selection.

 Additionally, some store managers thought that the impact of signage components on customer behavior was insufficient compared to the associated workload, and deprioritized it. Attempts were made to highlight the value of the signage through sharing preliminary results of the RCT and emphasizing the importance of implementing each component for the entire trial period. However improvements were limited.

 Finally, some store managers disagreed with the HFI’s overall design philosophy. They found it too “passive” or “subtle” for effective health promotion, which negatively affected their motivation for the HFI. The solution to these issues primarily involved enhancing project communication: The regional managers supervising problematic stores were asked to reiterate the importance of implementing the HFI to these managers and the researchers reiterated the reasoning behind the HFI’s design. Mutually acceptable adjustments for HFI components were sought. While not all disagreements were resolved, implementation did generally improve in these stores.

 The second factor was the high valuation of *commercial success*. This was evident both in the stores and the wider organizational culture, with discussions about the HFI primarily revolving around commercial outcomes. Regional and store managers primarily discussed and evaluated the HFI (especially presentation components, due to the limited space available) in terms of profitability and opportunity costs. Due to the negative perception of healthy products in these terms, store managers often decided to instead present (better performing) unhealthy products. Finding effective solutions to these issues proved challenging. An attempt was made to let the store managers themselves choose more profitable healthy products, but most forgot or were too occupied. Lastly, one store manager was dissatisfied with the HFI as they had hoped it would help create a healthier appearance attracting more customers, and found the implemented components too subtle for this purpose. The researchers explained the reasons behind the HFI’s design, but it did not fully address the underlying concern.

###  External Factors

 The HFI was influenced by various external factors. One factor was the *consumer landscape*, where unhealthy products seemed to be in significantly higher demand than healthy ones. This influenced store employees’ beliefs about the popularity of healthy products. Secondly, *external obligations *such as supplier contracts forced stores to engage in practices that were contrary to the HFI’s goals, such as placing displays with unhealthy products. Lastly, the COVID-19 pandemic was a disruptive *external event*. It affected project preparations, impeded the training and briefing of store employees, led to employee shortages (due to illness), and added safety measures to the already high workload of store employees. These factors were unfortunately outside the influence of the stores and researchers to resolve.

## Discussion

 The aim of this study was to explore “which systemic factors produce implementation and sustainment barriers for HFIs, and how?” Through an RMA approach, an HFI in Dutch supermarkets was monitored for adherence issues. These were subsequently explored to identify barriers, underlying factors, and how these factors led to the encountered barriers. Below we discuss the main outcomes and how these compare to the literature, followed by theoretical considerations, and the study’s strengths and limitations.

###  Main Barriers

 We identified several important systemic factors that impeded implementation of HFI, some of which were structural: First, limited resources (employee and key actor availability, health knowledge, store space) constrained the capacity of stores to maintain the HFI. Second, suboptimal integration of the HFI into processes and systems increased workload. resulting in additional workload and frustrations. Third, stores were evaluated primarily on commercial outcomes. Fourth, healthy products are more challenging to sell (eg, shorter shelf-life, lower demand).

 Other barriers were cultural: They included negative beliefs about the HFI’s impact on commercial outcomes and dietary behavior. Concerns about the commercial viability of promoted products, which led to low trust in the HFI among store managers, also impeded the implementation of the HFI. Additionally, organizational decision-making was dominated by a frame in which activities were primarily evaluated on their contribution to commercial success. As a result, store managers prioritized what they perceived as most beneficial to their commercial outcomes (regular store activities). This left limited resources for the HFI, thus harming its implementation and sustainment. This issue was further compounded by factors relating to the design and the implementation process of the intervention, and external factors.

 These findings are generally consistent with the broader literature: A recent examination of reviews identified resource constraints (time, people, space), low (perceived) demand for healthy products, friction between commercial goals and health promotion, external influences such as suppliers, and process-related such as low organizational support as barriers for HFIs.^6^ Another study, that examined an HFI maintained by store managers and employees, encountered similar concerns regarding the commercial impact of the HFI and potential waste risks, difficulties in finding the optimal stock ranges for promoted products, and interference from suppliers.^11^ Notably, employee training, communication with the stores, and organizational support in this study were better compared to our own. This contributed to more motivated managers and employees and greater priority being given to the HFI. Another facilitating factor may have been the shorter timespan of the study (12 weeks).^11^ Finally, a recent study mapped the systemic factors involved in making food store environments healthier, via explorative interviews with food retailers.^13^ It found that resistance to an HFI would decrease over time as positive results are demonstrated,^13^ which we failed to do in our study. Furthermore, multitudes of changes (eg, multiple components) become increasingly difficult to maintain, due to compounding workload,^13^ as observed in our study. Additionally, organizational support, and the associated allocation of sufficient resources were again noted as vital factors,^13^ the lack of which posed clear barriers in our study.

###  On Dealing with Systemic Factors

 Although this study examined systemic factors as one group, our findings illustrate that there are distinctions to be made in how they pose barriers, and how they could be addressed.

 Several structural factors, primarily IT systems, processes, or infrastructure, produced barriers due to the HFI not being integrated successfully in these structures, or unrelated failures (eg, bugs) within these structures. An example is the bugs in the price management system which affected the pricing component. The solution was usually adjustments to the factor (eg, resolving bugs) or HFI (eg, adjusting planning) to improve integration. These solutions are generally under the direct control of the organizational actors and researchers and desirable for the organization (eg, reducing workload/errors). Therefore, addressing these factors was often quick and relatively straightforward. Such factors posed “quick wins” for the project,^30^ and helped maintain a sense of momentum for the store and organizational actors, benefitting the sustainment of the HFI.^31,32^

 In contrast, cultural factors, particularly the high valuation of commercial success, and associated beliefs on how it is best pursued, produced more complicated barriers: Store and regional managers in particular often perceived the HFI as inconsequential or even detrimental to commercial outcomes. This is facilitated by the translation of the abstract ideal of commercial success into explicit structures,^4,33^ eg, performance metrics. These structures in turn put pressure on the stores to deprioritize the HFI. To solve this issue, commercial success needs to become less valued, or the HFI needs to be perceived as complementary to this value.

 Unfortunately, doing so is relatively complicated. This is illustrated by the fact that the potential issues posed by these values and beliefs were identified in advance during the co-creative development of the HFI.^8,19,34^ The idea that unhealthy products are more profitable than healthy ones seems highly persistent, likely due to its historical success in driving sales.^15^ As such, hard proof of positive results is likely required to overcome these beliefs,^13^ which this study failed to provide in part due to difficulties in obtaining the necessary data. In summary, it seems that, to make HFIs easier to implement and sustain in food store settings, concrete examples of such interventions contributing to the “success” of these stores are needed.

###  On Achieving Change

 To develop more positive examples of successful HFIs, and drive change on a broader level, a strategy is needed that goes beyond individual interventions. The Transition Management Framework is an example of such a strategy^32^: It prescribes coordinated experimentation across contexts across the system, accumulating evidence, inspiration, and momentum. Central to this are so-called “frontrunners”: people within the system (eg, store managers) who are motivated to make a change, and who act as insiders and catalysts for change within the system. Identifying and involving these frontrunners is crucial to setting up and spreading HFIs.^32^

 In transition management, “niches” play an important role in experimentation. These are isolated spaces in which new ideas can be developed. HFIs, aiming to change the system, face challenges and may be pressured to conform,^19^ risking dilution, or perversion to the goals of the system, such as greenwashing.^35,36^ A protective niche, for example a store exempt from usual metrics, shields the HFI until it has been developed enough, and has sufficient evidence behind it, to challenge the system on its own terms. This study illustrates what can happen when an HFI is not sufficiently protected: it will be challenged and obstructed by various systemic influences that attempt to reshape it to fit the existing cultures and structures, and otherwise reject it. This highlights the challenge of conducting HFI research within the dominant food store system. Exploring alternatives like empowering or establishing new, health promoting retail formats, could be valuable.^37^

 Transition management considers variation a vital aspect of developing new ideas. It provides space for adaptation to changing circumstances, and enables the emergence of stronger ideas through continuous selection based on performance.^32,38,39^ In this study, variation was constrained due to the RCT’s need for consistency. Ideally, underperforming HFI components would be adapted to improve over time. In such a scenario, the strengths of the RMA approach would be better served. However, these restrictions on variation also limited the RMA approach in exploring more rigorous solutions such as adjusting certain components – although the method remained valuable for identifying underlying systemic factors. For future applications of RMA, we recommend a more flexible setup^40^ whereby an initial HFI version is implemented, monitored, and adapted over an extended period. Subsequently, the adapted version can be rigorously evaluated through methods such as an RCT, bridging the observed gap between reported impact and real-world feasibility.^41^

 Finally, it is important to note that RMA does not need to end once the facilitating researchers step away, but can be taken over by the implementing organization, as an optimization tool. However, this does require there to be organizational support for such efforts, and motivated people who can be trained to coordinate the process, which may not always be the case.

###  Strengths and limitations

 The strengths of the study were:

1. The study had a relatively long timeframe as a trial, which facilitated the exploration and evaluation of barriers and underlying factors. However, as a niche experiment, the timeframe could be considered short, thus limiting the time to (potentially) address more deeply embedded barriers and factors.
2. The project duration allowed for repeated interactions with the same group of actors, which facilitated the building of rapport and mutual understanding, resulting in more in-depth data collection.
3. The mixed-methods approach reduced the role of individual biases among store actors regarding which implementation and sustainment barriers and underlying factors were explored, as quantitative monitoring outcomes indicated those components which experienced major barriers. These barriers could then specifically be explored in depth through qualitative discussions.
4. The study examined a wide range of popular health intervention strategies out in real-world supermarkets. This makes our findings highly representative of real-world settings, and relevant for a variety of HFI designs.

The study had three main weaknesses:

1. The range of interviewed actors was relatively limited, thus introducing potential biases in the data. The customers who interacted with the HFI were not included in the study. The central office actors did not include representatives from every relevant department, because we encountered no issues related to their domains, and these actors were extremely busy. When issues specific to an actor were noticed, this actor would be contacted by researchers or already involved central office actors.
2. Data collection was performed by multiple individuals, which may have introduced inconsistencies. These individuals discussed the data collection process in advance, and evaluated outcomes afterward, to minimize biases.
3. Interviews were conducted by video and telephone calls, which may have reduced their depth. Unfortunately, this was a result of the COVID-19 restrictions in place at that time.

## Conclusion

 This study explored the systemic factors that produce barriers for HFI implementation and sustainment, and how they do so. For this purpose, the study applied a systems innovation perspective to the issue of HFI implementation and sustainment barriers, through the application of an RMA approach. The study illustrates the potential value of RMA for evaluating and developing HFIs. The dominant valuation of commercial success and associated beliefs on how this is best achieved present major drivers of the promotion of unhealthy products. These values are codified in processes and rules, such as performance metrics, which motivate store managers to prioritize the pursuit of commercial success over health promotion. This pressure, in combination with limited resources, and substantial friction between an HFI and the regular activities in a store, lead to priority being given to these regular, unhealthy-products-promoting activities. This leaves limited time and motivation for maintenance of the HFI. These issues are exacerbated by other factors: External factors, can further constrain resources and reinforce problematic beliefs. Intervention-related factors may increase workload or friction. Finally, process-related factors can frustrate the exchange of important information and reduce (perceived) support for the intervention. Based on these findings and a systems innovation perspective we discussed several considerations for future research. These included the role of frontrunners in developing support and space for HFIs, the importance of establishing safe spaces for the evaluation and development of HFIs, and the value of variation in the development of HFIs. These results can be used for the development of future HFIs.

## Acknowledgements

 This work was part of the SUPREME NUDGE project. We also wish to thank Anouk ter Balkt for her assistance with data collection, and Lotte Levelt for her help in editing the final manuscript.

## Ethical issues

 This study was subjected to the self-check of the Ethics Review Committee of the VU University Faculty of Science, and was deemed to comply with the code of ethics and required no further review.^42^ Interviewees were informed about the study design and goals and their right to withdraw participation or withhold sensitive information. We asked for consent to record the interview and used the anonymized transcript for academic publications. All participants consented.

## Competing interests

 Cédric N.H. Middel reports grants from Dutch Heart Foundation, grants from ZonMW, grants from NWO, during the conduct of the study; and at the time of the study, the authors were in collaboration with the supermarket organisation described in this study. The organisation did not influence, alter, or limit our findings and their interpretation in any form.

## Funding

 This work was supported by the SUPREME NUDGE project (CVON2016–04), which is funded by the Dutch Heart Foundation and ZonMW. JDM’s work was funded by an NWO VENI grant on “Making the healthy choice easier—role of the local food environment” (grant number 451-17-032). These funders were uninvolved in the work presented in this paper.

## Supplementary files

Supplementary file 1. Monitoring Checklist.

Supplementary file 2. Adherence Scores.

Supplementary file 3. Barriers and Solutions Overview.
