# Supplementary material for: A Systems Innovation Perspective on Implementation and Sustainment Barriers for Healthy Food Store Interventions: A Reflexive Monitoring in Action Study in Dutch Supermarkets
Source: Int J Health Policy Manag. 2024 May 12;13:8036. doi: 10.34172/ijhpm.2024.8036 (PMC11270604; doi:10.34172/ijhpm.2024.8036)
Supplement: Supplementary file 1 — Monitoring Checklist. [file ijhpm-13-8036-s001.pdf]

Article title: A Systems Innovation Perspective on Implementation and Sustainment Barriers for Healthy Food Store Interventions: A Reflexive Monitoring in Action Study in Dutch Supermarkets

Journal name: International Journal of Health Policy and Management (IJHPM)

Authors' information: Cédric N.H. Middel[1,2,3,4]*, Tjerk Jan Schuitmaker-Warnaar[1], Joreintje D. Mackenbach[2,3,4], Jacqueline E.W. Broerse[1]

[1]Athena Institute, Faculty of Science, Vrije Universiteit Amsterdam, Amsterdam, The Netherlands.

[2]Department of Epidemiology and Data Science, Amsterdam University Medical Centers, Vrije Universiteit Amsterdam, Amsterdam, The Netherlands.

[3]Upstream Team, www.upstreamteam.nl, Amsterdam University Medical Centers, Amsterdam, The Netherlands.

[4]Amsterdam Public Health, Health Behaviors and Chronic Diseases, Amsterdam, The Netherlands.

***Correspondence to:** Cédric N.H. Middel; Email: c.n.h.middel@amsterdamumc.nl

Citation: Middel CNH, Schuitmaker-Warnaar TJ, Mackenbach JD, Broerse JEW. A systems innovation perspective on implementation and sustainment barriers for healthy food store interventions: a reflexive monitoring in action study in Dutch supermarkets. Int J Health Policy Manag. 2024;13:8036. doi:10.34172/ijhpm.2024.8036

Supplementary file 1. Monitoring Checklist

Shown below is an empty version of the checklist tool used for the monitoring visits, translated from its original language (Dutch).

Store:

Date:

Researcher:

Intervention	Intended products		Scored characteristic	5-point scale	Remarks
**Head-shelf presentation**<br><br>(Complete head shelf, products presented with uniform communication style, corresponding to the assigned shelf-tags)	1.		Correct position	1 2 3 4 5	
	2.				
	3.				
	4.		Correct products	1 2 3 4 5	
	5.				
	6.		Undamaged, clean, readable	1 2 3 4 5	
	7.				
	8.				
**Basket presentation**<br><br>(at the checkout or distributed throughout aisles, communication should have a nudge theme)	1.		Correct position	1 2 3 4 5	
	2.		Correct products	1 2 3 4 5	
	3.		Undamaged, clean, readable	1 2 3 4 5	
**Shelf tags**<br><br>(at all healthy products for each product group)	**Percentage correct, per product group**		Correct products	1 2 3 4 5	
	**1.** Pre-cut fruits, vegetables and potatoes				
	**2.** Fresh fruits, vegetables and potatoes				
	**3.** Biscuits and crackers				
	**4.** Bread spreads				
	**5.** Water and soft drinks				
	**6.** Butter				
	**7.** Frozen vegetables				
	**8.** Preserved vegetables				
	**9.** Long-life bread				
	**10.** Long-life dairy				
	**11.** Frozen fruits				
	**12.** Cheese				
	**13.** Luxury nuts				
	**14.** Peanuts		Undamaged, clean, readable	1 2 3 4 5	
	**15.** Breakfast cereals				
	**16.** Rice and pasta				

	**17.** Sauces and sours					
	**18.** Thee					
	**19.** Fresh dairy					
	**20.** Frozen fish					
	**21.** Fresh fish					
	**22.** Preserved fish					
**Shelf cards**	**Intended products**					
(at a specific product for each product group)	1.			Correct position	1 2 3 4 5	
	2.					
	3.					
	4.					
	5.					
	6.					
	7.			Correct products	1 2 3 4 5	
	8.					
	9.					
	10.					
	11.					
	12.					
	13.					
	14.			Undamaged, clean, readable	1 2 3 4 5	
	15.					
	16.					
	17.					
	18.					
	19.					
	20.					
	21.					
	22.					
**Intervention**	**Information**			**Scored characteristic**	**5-point scale**	**Remarks**
**Shelf position**<br><br>(shelf positions adjusted for each product group)	Rice and pasta;<br><br>Biscuits and crackers;<br><br>Breakfast cereals			Correct position	1 2 3 4 5	
**Check-out presentation**<br><br>(unhealthy products replaced with healthy products at	At the first check-out			Correct position	1 2 3 4 5	
				Correct products	1 2 3 4 5	

check-out impulse shelf)					
**Posters**	At fruits and vegetable island		Correct position	1 2 3 4 5	
(standing poster position between healthy products)			Undamaged, clean, readable	1 2 3 4 5	
**Banners**	Biscuits		Correct position	1 2 3 4 5	
	Legumes				
(banners on shelves carrying the following product groups)	Breakfast cereals				
	Pasta				
	Rice		Undamaged, clean, readable	1 2 3 4 5	
**Feedback strip**	bread		Correct position	1 2 3 4 5	
(Strip beneath healthy products for the following product groups)	Vegetables (uncooled snack vegetables)		Correct products	1 2 3 4 5	
	Fish				
	Vegetables (cooled lettuce)		Undamaged, clean, readable	1 2 3 4 5	
**Cart handles**	Stickers on shopping cart handle		Correct position	1 2 3 4 5	
			Undamaged, clean, readable	1 2 3 4 5	
**Cart boards**	Boards on the front of shopping carts		Correct position	1 2 3 4 5	
			Undamaged, clean, readable	1 2 3 4 5	
**Shopping basket placemats**	Placemats on the bottom of shopping baskets		Correct position	1 2 3 4 5	
			Undamaged, clean, readable	1 2 3 4 5	
**Check-out divider bars**	50% of the divider bars must carry intervention communication		Correct position	1 2 3 4 5	
			Undamaged, clean, readable	1 2 3 4 5	
**Price mutations**	Have the prices been adjusted correctly?		Correct prices	1 2 3 4 5	
**Price cards**	Are there 'temporarily reduced price' tags attached to the discounted products?		Correct products	1 2 3 4 5	
			Undamaged, clean, readable	1 2 3 4 5	
