# Supplementary material for: A Systems Innovation Perspective on Implementation and Sustainment Barriers for Healthy Food Store Interventions: A Reflexive Monitoring in Action Study in Dutch Supermarkets
Source: Int J Health Policy Manag. 2024 May 12;13:8036. doi: 10.34172/ijhpm.2024.8036 (PMC11270604; doi:10.34172/ijhpm.2024.8036)
Supplement: Supplementary file 2 — Adherence Scores. [file ijhpm-13-8036-s002.pdf]

Article title: A Systems Innovation Perspective on Implementation and Sustainment Barriers for Healthy Food Store Interventions: A Reflexive Monitoring in Action Study in Dutch Supermarkets
Journal name: International Journal of Health Policy and Management (IJHPM)
Authors' information: Cédric N.H. Middel[1,2,3,4]*, Tjerk Jan Schuitmaker-Warnaar[1], Joreintje D. Mackenbach[2,3,4], Jacqueline E.W. Broerse[1]
[1]Athena Institute, Faculty of Science, Vrije Universiteit Amsterdam, Amsterdam, The Netherlands.
[2]Department of Epidemiology and Data Science, Amsterdam University Medical Centers, Vrije Universiteit Amsterdam, Amsterdam, The Netherlands.
[3]Upstream Team, www.upstreamteam.nl, Amsterdam University Medical Centers, Amsterdam, The Netherlands.
[4]Amsterdam Public Health, Health Behaviors and Chronic Diseases, Amsterdam, The Netherlands.
***Correspondence to:** Cédric N.H. Middel; Email: c.n.h.middel@amsterdamumc.nl
Citation: Middel CNH, Schuitmaker-Warnaar TJ, Mackenbach JD, Broerse JEW. A systems innovation perspective on implementation and sustainment barriers for healthy food store interventions: a reflexive monitoring in action study in Dutch supermarkets. Int J Health Policy Manag. 2024;13:8036. doi:10.34172/ijhpm.2024.8036
Supplementary file 2. Adherence Scores

For each HFI component, the checklist lists the appropriate options out of the following characteristics:

1) 'is the correct product promoted?'
2) 'is it in the correct position?'
3) 'are prices adjusted correctly?'
4) 'is it undamaged, clean, and readable?'.

Each characteristic was individually scored on a 5-point Likert scale, with 1 indicating approximately 0-20% adherence, and 5 representing 80-100% adherence (the acceptable range). A score of X means the intervention was (temporarily) discontinued by the interventionists.

The price mutation and price card components were stopped for two months during the implementation of a new price-management system. Shopping basket placemats were only implemented after two months due to baskets not being in use before then. In store C, the banner component was not implemented due to the shelves not being able to carry banners. In store D, the cart handles were not implemented due to the shopping carts having a different type of handles, unsuited for this component.

Table 1. This table shows the monitoring outcomes for store A

HFI component		Characteristic	Weeks since implementation											
			2	4	6	8	10	12	16	20	25	30	36	46
Correct product presentation	Check-out presentation	Correct position	1	1	3	5	3	1	1	1	1	1	1	1
		Correct product	1	1	3	5	3	1	1	1	1	1	1	1
	Shelf position	Correct position	3	5	5	5	5	5	5	5	5	5	5	5
	Head-shelf presentation	Correct position	5	5	5	5	5	5	5	5	5	5	5	5
		Correct product	4	5	5	5	5	5	5	5	5	5	5	5
		Undamaged, clean, readable	5	5	5	5	4	3	5	5	5	5	5	4
	Basket presentation	Correct position	1	5	5	3	2	3	5	1	1	5	1	1
		Correct product	1	5	5	5	5	5	5	1	1	4	1	1
		Undamaged, clean, readable	1	3	5	5	3	4	5	1	1	5	1	1
Signage	Shelf tags	Correct position	5	5	5	5	5	5	5	5	4	5	5	5
		Undamaged, clean, readable	5	5	5	5	5	5	5	5	4	5	5	5
	Posters	Correct position	5	5	5	5	5	5	5	5	5	5	5	5
		Undamaged, clean, readable	5	5	5	5	5	5	5	5	5	5	5	5
	Feedback strip	Correct position	5	5	5	5	5	4	4	5	5	5	5	5
		Correct product	5	5	5	5	5	5	5	5	5	5	5	5
		Undamaged, clean, readable	5	5	5	5	5	5	5	5	5	5	5	5
	Banners	Correct position	3	3	3	3	3	3	3	3	5	5	5	5
		Undamaged, clean, readable	5	5	5	5	5	5	5	5	5	5	5	3
	Check-out divider bars	Correct position	5	5	5	5	5	1	1	5	4	5	2	1
		Undamaged, clean, readable	5	5	5	5	5	1	1	5	5	5	5	1
	Shopping basket placemats	Correct position	X	X	X	X	4	3	3	4	1	1	1	3
		Undamaged, clean, readable	X	X	X	X	3	3	3	5	5	4	1	3
	Cart boards	Correct position	5	5	5	5	5	5	5	5	4	5	5	4
		Undamaged, clean, readable	5	5	5	4	4	5	5	5	5	5	5	5
	Cart handles	Correct position	5	5	5	5	4	5	5	5	5	5	5	4
		Undamaged, clean, readable	5	5	5	5	4	5	5	5	5	5	5	5
	Shelf cards	Correct position	4	5	3	1	3	3	5	5	5	5	5	3
		Correct product	4	5	1	1	1	3	5	5	1	5	1	1
		Undamaged, clean, readable	4	5	5	1	5	5	5	5	5	5	5	4
	Price cards	Correct position	3	1	1	1	1	3	X	X	1	3	1	1
		Undamaged, clean, readable	5	1	1	1	1	5	X	X	1	5	1	1
Pricing	Price mutations	Correct price	4	2	1	1	1	4	X	X	4	5	5	5

Table 2. This table shows the monitoring outcomes for store B

HFI component		Characteristic	Weeks since implementation											
			2	4	6	8	10	12	16	20	25	30	36	46
Correct product presentation	Check-out presentation	Correct position	1	1	5	5	5	5	5	5	5	5	5	5
		Correct product	1	1	5	5	5	5	5	5	5	5	5	3
	Shelf position	Correct position	3	3	5	5	5	5	5	5	5	5	5	5
	Head-shelf presentation	Correct position	1	1	3	3	5	5	5	5	5	5	5	5
		Correct product	1	1	5	5	5	5	1	5	5	5	5	5
		Undamaged, clean, readable	1	1	2	5	5	5	5	5	5	5	5	5
	Basket presentation	Correct position	1	1	5	5	5	5	4	5	5	4	2	5
		Correct product	1	1	5	5	1	4	4	5	2	4	2	5
		Undamaged, clean, readable	1	1	4	5	5	5	5	5	5	5	5	4
Signage	Shelf tags	Correct position	4	5	5	5	4	5	5	5	5	5	5	5
		Undamaged, clean, readable	5	5	5	5	5	5	5	5	5	5	5	5
	Posters	Correct position	5	5	5	5	5	5	5	5	5	5	5	5
		Undamaged, clean, readable	5	5	5	5	5	5	5	5	5	5	5	5
	Feedback strip	Correct position	5	5	5	5	5	5	3	3	3	5	4	5
		Correct product	5	5	5	5	5	5	5	5	5	5	5	5
		Undamaged, clean, readable	5	5	5	5	5	5	5	4	5	5	5	5
	Banners	Correct position	1	1	1	1	1	1	4	4	4	5	5	5
		Undamaged, clean, readable	1	1	1	1	1	1	5	5	5	5	5	5
	Check-out divider bars	Correct position	5	5	5	5	5	5	5	5	4	5	5	5
		Undamaged, clean, readable	5	5	5	5	5	5	5	5	5	5	5	5
	Shopping basket placemats	Correct position	X	X	X	X	X	1	3	3	2	3	3	5
		Undamaged, clean, readable	X	X	X	X	X	1	5	3	3	4	5	4
	Cart boards	Correct position	5	5	5	5	5	5	5	5	5	5	5	5
		Undamaged, clean, readable	5	5	5	5	5	5	5	5	5	5	5	5
	Cart handles	Correct position	4	5	5	5	5	5	5	5	5	5	4	5
		Undamaged, clean, readable	5	5	5	5	5	5	5	5	5	5	5	5
	Shelf cards	Correct position	5	5	5	4	5	4	4	5	5	5	4	2
		Correct product	5	5	3	2	5	4	1	5	1	1	1	1
		Undamaged, clean, readable	5	5	4	4	3	5	5	5	5	5	5	4
	Price cards	Correct position	1	1	3	3	3	3	X	X	1	1	1	1
		Undamaged, clean, readable	1	1	5	5	5	5	X	X	1	5	5	1
Pricing	Price mutations	Correct price	1	2	4	4	3	5	X	X	4	5	5	5

Table 3. This table shows the monitoring outcomes for store C

HFI component		Characteristic	Weeks since implementation										
			**2**	**4**	**6**	**8**	**12**	**16**	**21**	**24**	**29**	**39**	**48**
Correct product presentation	Check-out presentation	Correct position	1	1	1	5	1	1	1	1	5	5	5
		Correct product	1	1	1	5	1	1	1	1	5	3	5
	Shelf position	Correct position	1	2	4	4	4	5	5	5	5	5	5
	Head-shelf presentation	Correct position	1	1	5	5	5	5	5	5	5	5	5
		Correct product	1	1	5	4	1	5	1	1	5	1	1
		Undamaged, clean, readable	1	1	5	5	5	3	5	5	5	5	2
	Basket presentation	Correct position	1	1	5	5	5	1	1	1	1	1	1
		Correct product	1	1	5	5	3	1	1	1	1	1	1
		Undamaged, clean, readable	1	1	5	5	3	1	1	1	1	1	1
Signage	Shelf tags	Correct position	5	5	5	5	5	5	5	5	5	5	5
		Undamaged, clean, readable	5	5	5	5	5	5	5	5	5	5	5
	Posters	Correct position	5	5	5	1	5	5	5	5	5	5	5
		Undamaged, clean, readable	5	5	5	1	5	5	5	5	5	5	5
	Feedback strip	Correct position	5	5	5	3	4	4	5	5	5	5	4
		Correct product	5	5	5	5	5	5	5	5	5	5	5
		Undamaged, clean, readable	5	5	5	5	5	5	5	5	5	5	5
	Banners	Correct position	X	X	X	X	X	X	X	X	X	X	X
		Undamaged, clean, readable	X	X	X	X	X	X	X	X	X	X	X
	Check-out divider bars	Correct position	5	5	1	1	2	1	1	1	5	1	1
		Undamaged, clean, readable	5	5	1	1	4	5	1	1	5	1	1
	Shopping basket placemats	Correct position	X	X	X	X	X	1	1	1	1	1	1
		Undamaged, clean, readable	X	X	X	X	X	1	1	1	1	1	1
	Cart boards	Correct position	5	5	5	5	5	5	4	4	5	5	4
		Undamaged, clean, readable	5	5	3	3	3	5	4	5	5	5	5
	Cart handles	Correct position	5	5	5	5	4	4	5	5	5	5	5
		Undamaged, clean, readable	5	5	5	5	5	4	5	5	5	5	5
	Shelf cards	Correct position	5	5	5	4	3	3	5	2	3	1	1
		Correct product	5	5	1	1	1	1	1	1	1	1	1
		Undamaged, clean, readable	5	4	4	5	4	5	5	4	4	1	1
	Price cards	Correct position	5	1	1	4	X	X	1	1	1	1	1
		Undamaged, clean, readable	5	1	1	5	X	X	1	1	1	1	1
Pricing	Price mutations	Correct price	4	3	2	3	X	X	1	1	5	2	4

Table 4. This table shows the monitoring outcomes for store D

HFI component		Characteristic	Weeks since implementation										
			**2**	**4**	**6**	**8**	**12**	**16**	**21**	**24**	**29**	**39**	**48**
Correct product presentation	Check-out presentation	Correct position	1	1	1	1	1	1	1	1	3	1	1
		Correct product	1	1	1	1	1	1	1	1	1	1	1
	Shelf position	Correct position	4	4	4	5	4	5	5	5	5	5	5
	Head-shelf presentation	Correct position	1	4	3	1	2	5	5	5	3	1	2
		Correct product	1	4	3	1	1	5	5	5	1	1	2
		Undamaged, clean, readable	1	4	4	1	2	4	4	5	3	1	2
	Basket presentation	Correct position	1	1	1	1	1	4	4	2	1	5	2
		Correct product	1	1	1	1	1	5	4	4	1	5	4
		Undamaged, clean, readable	1	1	1	1	1	3	3	5	1	5	2
Signage	Shelf tags	Correct position	5	5	5	5	5	5	5	5	5	5	5
		Undamaged, clean, readable	5	5	5	5	5	5	5	5	5	5	5
	Posters	Correct position	1	1	1	1	1	1	5	5	5	5	5
		Undamaged, clean, readable	1	1	1	1	1	1	5	5	5	5	5
	Feedback strip	Correct position	5	5	5	4	3	4	4	5	5	4	5
		Correct product	5	5	5	4	3	5	5	5	5	5	5
		Undamaged, clean, readable	5	5	5	4	5	5	5	5	5	5	5
	Banners	Correct position	1	1	1	1	1	2	4	5	5	5	5
		Undamaged, clean, readable	1	1	1	1	1	5	5	5	5	5	5
	Check-out divider bars	Correct position	5	5	5	1	1	5	4	5	1	1	1
		Undamaged, clean, readable	5	5	5	1	1	5	5	5	1	1	1
	Shopping basket placemats	Correct position	X	X	X	3	1	4	2	2	2	2	1
		Undamaged, clean, readable	X	X	X	2	1	4	3	4	3	3	3
	Cart boards	Correct position	5	5	5	5	5	5	5	5	5	5	5
		Undamaged, clean, readable	5	3	3	3	3	5	5	5	5	5	3
	Cart handles	Correct position	X	X	X	X	X	X	X	X	X	X	X
		Undamaged, clean, readable	X	X	X	X	X	X	X	X	X	X	X
	Shelf cards	Correct position	5	5	5	1	2	2	3	4	2	1	1
		Correct product	5	5	5	1	2	1	1	1	1	1	1
		Undamaged, clean, readable	5	4	3	1	5	5	5	5	4	1	1
	Price cards	Correct position	1	1	1	1	X	X	1	1	1	1	1
		Undamaged, clean, readable	1	1	1	1	X	X	1	5	1	1	1
Pricing	Price mutations	Correct price	1	1	1	1	X	X	1	2	1	5	3

Table 5. This table shows the monitoring outcomes for store E

**HFI component**		**Characteristic**	**Weeks since implementation**					
			**4**	**10**	**15**	**20**	**24**	**28**
Correct product presentation	Check-out presentation	Correct position	1	1	1	5	5	5
		Correct product	1	1	1	4	5	5
	Shelf position	Correct position	2	5	5	5	5	5
	Head-shelf presentation	Correct position	5	5	5	5	5	5
		Correct product	5	5	5	4	3	1
		Undamaged, clean, readable	5	5	5	5	5	5
	Basket presentation	Correct position	4	4	4	1	2	1
		Correct product	4	5	5	1	2	1
		Undamaged, clean, readable	5	5	5	1	1	1
Signage	Shelf tags	Correct position	5	5	5	5	5	5
		Undamaged, clean, readable	5	5	5	5	5	5
	Posters	Correct position	1	5	5	5	5	5
		Undamaged, clean, readable	1	5	5	5	5	5
	Feedback strip	Correct position	5	5	5	5	5	5
		Correct product	5	5	5	5	5	5
		Undamaged, clean, readable	5	5	5	5	5	5
	Banners	Correct position	5	5	5	5	5	5
		Undamaged, clean, readable	5	5	5	5	5	5
	Check-out divider bars	Correct position	5	5	5	5	5	5
		Undamaged, clean, readable	5	5	5	5	5	5
	Shopping basket placemats	Correct position	3	3	2	2	2	3
		Undamaged, clean, readable	5	5	5	5	4	4
	Cart boards	Correct position	5	5	5	5	4	5
		Undamaged, clean, readable	5	5	5	4	5	5
	Cart handles	Correct position	5	5	5	5	5	5
		Undamaged, clean, readable	5	5	5	5	5	5
	Shelf cards	Correct position	5	5	3	5	2	1
		Correct product	5	1	1	1	1	1
		Undamaged, clean, readable	5	5	4	4	4	1
	Price cards	Correct position	5	4	1	1	1	1
		Undamaged, clean, readable	5	5	1	1	1	1
Pricing	Price mutations	Correct price	5	5	5	4	2	5

Table 6. This table shows the monitoring outcomes for store F

HFI component		Characteristic	Weeks since implementation					
			**4**	**10**	**15**	**20**	**24**	**28**
Correct product presentation	Check-out presentation	Correct position	5	5	5	5	5	1
		Correct product	5	5	3	3	3	1
	Shelf position	Correct position	5	5	5	5	5	5
	Head-shelf presentation	Correct position	5	5	5	5	5	1
		Correct product	5	5	5	5	1	1
		Undamaged, clean, readable	5	3	5	5	3	1
	Basket presentation	Correct position	1	1	1	1	1	1
		Correct product	1	1	1	1	1	1
		Undamaged, clean, readable	1	1	1	1	1	1
Signage	Shelf tags	Correct position	5	5	5	5	5	5
		Undamaged, clean, readable	5	5	5	5	5	5
	Posters	Correct position	1	1	1	1	1	4
		Undamaged, clean, readable	1	1	1	1	1	5
	Feedback strip	Correct position	5	4	4	4	4	4
		Correct product	5	5	5	5	5	5
		Undamaged, clean, readable	5	5	5	5	5	5
	Banners	Correct position	1	1	1	1	1	1
		Undamaged, clean, readable	1	1	1	1	1	1
	Check-out divider bars	Correct position	5	1	1	1	1	1
		Undamaged, clean, readable	5	1	1	1	1	1
	Shopping basket placemats	Correct position	3	3	2	2	2	2
		Undamaged, clean, readable	5	5	5	5	4	4
	Cart boards	Correct position	5	5	5	5	4	4
		Undamaged, clean, readable	5	4	5	5	5	5
	Cart handles	Correct position	5	5	5	5	4	4
		Undamaged, clean, readable	5	5	5	5	5	5
	Shelf cards	Correct position	5	4	3	4	3	1
		Correct product	5	1	1	1	1	1
		Undamaged, clean, readable	5	5	4	4	4	1
	Price cards	Correct position	1	1	1	1	1	1
		Undamaged, clean, readable	1	1	1	1	1	1
Pricing	Price mutations	Correct price	5	5	5	5	5	5
