# Supplementary material for: A Systems Innovation Perspective on Implementation and Sustainment Barriers for Healthy Food Store Interventions: A Reflexive Monitoring in Action Study in Dutch Supermarkets
Source: Int J Health Policy Manag. 2024 May 12;13:8036. doi: 10.34172/ijhpm.2024.8036 (PMC11270604; doi:10.34172/ijhpm.2024.8036)
Supplement: Supplementary file 3 — Barriers and Solutions Overview. [file ijhpm-13-8036-s003.pdf]

Article title: A Systems Innovation Perspective on Implementation and Sustainment Barriers for Healthy Food Store Interventions: A Reflexive Monitoring in Action Study in Dutch Supermarkets

Journal name: International Journal of Health Policy and Management (IJHPM)

Authors' information: Cédric N.H. Middel[1,2,3,4]*, Tjerk Jan Schuitmaker-Warnaar[1], Joreintje D. Mackenbach[2,3,4], Jacqueline E.W. Broerse[1]

[1]Athena Institute, Faculty of Science, Vrije Universiteit Amsterdam, Amsterdam, The Netherlands.

[2]Department of Epidemiology and Data Science, Amsterdam University Medical Centers, Vrije Universiteit Amsterdam, Amsterdam, The Netherlands.

[3]Upstream Team, www.upstreamteam.nl, Amsterdam University Medical Centers, Amsterdam, The Netherlands.

[4]Amsterdam Public Health, Health Behaviors and Chronic Diseases, Amsterdam, The Netherlands.

***Correspondence to:** Cédric N.H. Middel; Email: c.n.h.middel@amsterdamumc.nl

Citation: Middel CNH, Schuitmaker-Warnaar TJ, Mackenbach JD, Broerse JEW. A systems innovation perspective on implementation and sustainment barriers for healthy food store interventions: a reflexive monitoring in action study in Dutch supermarkets. Int J Health Policy Manag. 2024;13:8036. doi:10.34172/ijhpm.2024.8036

Supplementary file 3. Barriers and Solutions Overview

The table below provides a comprehensive overview of all factors identified in this study to play a direct or indirect role in producing barriers for the implementation and sustainment of the HFI. Factors are categorized under the different domains of the theoretical framework: Intervention; Implementation Process; Store Practice, Culture, Structure, Organization Practice, Culture, Structure; External Factors. A distinction is made between the systemic factors which are part of the store system ('store system practice, structure and culture') and those that are not directly linked to the stores, but rather the overarching organization ('organization system practice, structure and culture').

The 'affected HFI components' column lists which components were affected by a factor in terms of their implementation or sustainment. Brief descriptions of these components are provided in the main paper (Table 1). The 'solutions' column specifies solutions used to address barriers. Occasionally, no feasible solution could be found.

Factor	Description	Affected HFI components	Solutions
**Intervention**			
Product Focus	Products promoted by presentation components were often not traditionally used in such presentations, which led store actors to lack relevant knowledge and understanding of which products would be profitable, how the product would perform, and how much should be stocked	Check-out presentation<br>Shelf positions<br>Head-shelf presentation<br>Basket presentations	Where possible in-depth information on products (e.g., profit margins) was provided, and store actors could provide input on products selected and amount of stock needed
Workload	Several dynamic components involved substantial (often fluctuating) amounts of work for stores, which was difficult to plan around, and carry out with the ongoing employee shortages	Shelf cards<br>Price cards<br>Price mutations	Changes were made to components, to reduce workload where possible, e.g., through integration in existing systems and processes, and stores received a monthly prognosis for expected work.
**Factor**	**Description**	**Affected HFI components**	**Solutions**
**Implementation process**			
Explanation	Several store managers perceived a lack of explanation from within the organization regarding the components, and overall project, in terms of tasks, goals and expectations (e.g., compensation in performance metrics for the impact of the HFI, needing to order some products manually)	General factor	Where possible, the researchers would immediately contact new store managers, to explain the project, and plan a visit to show and explain all components and associated work. If a store manager did not know that the HFI would be taken into account for their performance reviews, the researchers would ask central office actors to discuss this with them
	Store employees had received very little explanation or training regarding the HFI, due to COVID-19 related restrictions in place at the start of the trial	General factor	Educational materials were developed for the store employees, to explain the HFI, and the underlying ideas and goals, which could be presented in the employee-areas in stores.
Communication	Changes in store management were frequently not communicated to the researchers and central office actors, leading them to only find out when the old manager was nearly leaving.	General factor	*No feasible solution was found*
	Store managers missed or had trouble finding necessary information for the implementation and maintenance of HFI, due to it being shared through non-traditional channels, or through traditional channels, but on different moments than usual. When unable to find information easily, store managers would often ignore the component.	General factor	Where possible, components were integrated into automated and traditional systems and channels. Where this was not possible, the researchers sent monthly reminders, with all the important information.

	Information on the project or components was often not provided in an understandable format.	General factor	The researchers would discuss how information was presented when problems were noticed, and make adjustments to improve its legibility where possible
	Store managers often noted a lack of hours to perform component-related work, but did not communicate with the central office on how many additional hours they would need	General factor	Whenever store managers noted a lack of hours, the researchers would inquire how many more they would need, and communicate this to the central office
	There were no structural communication moments regarding the HFI between store actors, or between central office actors, which made coordination and exchanging experiences and learnings difficult.	General factor	Efforts were made to organize group sessions. However, store actors would often not show up, due to being busy or forgetting. Reaching the central office actors also meeting with central office actors. Eventually the meetings were deemed unfeasible and discontinued
	Store actors received no direct feedback regarding commercial or health-related outcomes of the HFI in their stores, due to the planned interface not being feasible to set up.	General factor	Store actors received a one-time summary of preliminary effects of the HFI on the sales of several products, in the last quarter of the trial
Organizational Support	In most stores, managers noted their showed limited engagement in carrying out HFI components. The managers believed this to be related to a lack of interest and understanding of the project and health promotion efforts in general	General factor	*No feasible solution was found*
	Store managers perceived limited engagement and support from the broader organization due to the lack of space they felt was given for focusing on the project. This lack of space was perceived due to pressure from other organizational metrics and activities pushed by the organization, which negatively affected store managers' motivation for the project	General factor	The organizational management sent a letter of appreciation and encouragement to the store managers for their work on the HFI, as most sources of pressure could not be resolved.
Integration	Initially, presentation components were not integrated in the space planning system. As a result, replenishment of stock was also not automatic, and store managers had to manually order those products.	Check-out presentation<br>Shelf positions<br>Head-shelf presentation<br>Basket presentations	The presentation components were integrated within the space planning system, flagged as specifically for the participating stores
	The planned moments for changing the products in dynamic presentation components did not always align with the usual moments at which stores restock or change presentations, leading them to forget.	Head-shelf presentation<br>Basket presentations	Where possible, the researchers attempted to align component work with regular practices, and where this was not possible, send reminders to the stores
	Stores usually replace signage at specific moments of the week. The signage for certain components was not always planned to be put up at this same moment, and the required information was not always available in advance of those planned moments	Shelf cards<br>Price cards	Where possible, the researchers attempted to align component work with regular practices, and where this was not possible, send reminders to the stores

	The stores' regular price changes were always sent out at specific moments of the week, which did not align with when intervention-related price changes came in. This led to store managers occasionally forgetting about the component-related price mutations	Price mutations	Where possible, the researchers attempted to align component work with regular practices, and where this was not possible, send reminders to the stores
Supply	Stores needed to order new materials themselves if they ran out, and often forgot to do so. Occasionally, certain necessary materials were also not available. When materials were not in the store, the component would often simply be ignored.	Check-out divider bars<br>Shopping basket placemats<br>Cart boards	In solution, the researchers would often have to remind the store actors to order these materials, and where to do so, occasionally doing it for them
**Factor**	**Description**	**Affected HFI components**	**Solutions**
	**Store system practices**		
Friction and Prioritization	When friction occurred between components and regular practices, store managers and employees frequently seemed to prioritize the regular practices, while disregarding the components. This was especially apparent for components which were less similar to regular practices, high workload, or for which information and/or materials were difficult to find.	General factor	*No feasible solution was found, researchers asked to the store actors to focus on components were deemed the most important, based on expected impact on dietary behaviors*
	Store managers were under substantial pressure to optimize their work around organizational metrics, and participate in organizationally promoted programs and reorganizations, which often conflicted with HFI components.	General factor	The organizational management to send a letter of appreciation and encouragement to the store managers for their work on the HFI, as most sources of pressure could not be resolved.
	Presentation components were often noted to take space from more commercially viable products which could be promoted instead	Head-shelf presentation<br>Basket presentations	Store actors provided feedback on products selected, and eventually could select options from a pre-approved list.
	Signage components were often accidentally removed when regular signage in the stores was changed.	Check-out divider bars	Central office actors added a specific line in the instructions for store signage, which specified an exception for HFI materials. This had limited effect
Customer (Inter)actions	Customers often (accidentally) damaged or threw away intervention signage while shopping	Shopping basket placemats<br>Shelf cards<br>Price cards	Signage was attached with adhesives, to prevent falling out/removal
	Store managers received negative (angry/frustrated/confused) reactions from customers regarding incorrect prices in the stores, as a result of the pricing component	Price mutations	The issues leading to incorrect prices were identified and resolved by central office actors
**Factor**	**Description**	**Affected HFI components**	**Solutions**
	**Store system structures**		

Human Resources	Stores had limited employees available for component implementation and maintenance	General factor	No solution could be found, stores were asked to prioritize certain components over others
	The organization was going through a major reorganization of the store management and employee positions, which included substantial training and learning for store employees and management	General factor	*No feasible solution was found*
Knowledge Resources	The majority of stores went through several changes in management over the duration of the component trial, leading to loss of general- and component-related knowledge and in these stores	General factor	*No feasible solution was found*
	Store managers lacked information on the relative profit margins of different products, making it difficult for them to select profitable products for component presentations when they were allowed to choose	Head-shelf presentation<br>Basket presentations	The list of approved healthy products was expanded with a column detailing the relative profit margins of the products
	Employees at the stores often lacked the knowledge/skills to independently assess whether (new) products could be considered healthy and thus should be marked with signage	Shelf tags	The organizational database was linked with a database supplied by the Netherlands Nutrition Centre, which had indications for which products could be considered 'healthy'
Space resources	There was limited presentation space in stores, leading presentation components to compete with other presentation practices	Check-out presentation<br>Head-shelf presentation<br>Basket presentations	Stores were offered additional presentation baskets if they had floor space, but none took up the offer
	Store shelves in one store were of an older model, unable to carry the banner component	Banners	*No feasible solution was found, the banner component was not implemented in affected stores*
Products	Store actors often disagreed with the products selected for presentation components, by central office actors, citing various characteristics (e.g., shelf life, size, price, profit margin, dietary function, popularity), which they perceived made the product commercially unviable for such use.	Check-out presentation<br>Head-shelf presentation<br>Basket presentations	Store actors provided feedback on products selected, and eventually could select options from a pre-approved list. Citing the lack of information on profit margins for these products they did not always make a selection. Such information was also added to the list.
	Some stores had a backlog of certain products (seasonal products, or unsold stock of previous component-promoted products) which they felt they needed to prioritize in presentation spaces, to avoid losses	Check-out presentation<br>Head-shelf presentation<br>Basket presentations	*No feasible solution was found*

	A number of products in the stores have standard prices displayed on their packaging, making price adjustments confusing to customers	Price mutations	The researchers maintained a list of which products had such printed prices, and excluded them form mutations.
**Factor**	**Description**	**Affected HFI components**	**Solutions**
	**Store system culture**		
Beliefs	Some store managers disagreed with the overall design philosophy of the HFI. They perceived the HFI to be too subtle to be effective for health promotion, or showing that the store was engaged in health promotion	General factor	Discussions were had with these store actors, and regional management, to explain the component designs, the importance of implementing them as planned, and find an acceptable middle ground in their implementation
	Store actors believed certain components would be detrimental to how their store would score on performance metrics due to being less profitable, or requiring additional work hours. The store managers also had concerns that this could negatively affect their personal evaluations and performance bonuses	Check-out presentation<br>Head-shelf presentation<br>Basket presentations<br>Shelf cards<br>Price cards<br>Price mutations	Central office actors promised that store managers' performance bonusses would not be negatively affected by the HFI, and additional work hours would be made available to the stores.
	Store managers indicated a lack of believe in the effectiveness or impact of the component on changing customers' dietary behaviors	Check-out presentation<br>Shelf positions<br>Shelf cards	The central office sent a message to the participating stores, with the (positive) preliminary effects of several components on the sales of a sample of products
	Store actors often held strong believes regarding which products were commercially feasible based on perceptions regarding what an effective price-range would be, dietary-functions of products, profitability, 'presenting attractively,' and demand.	Check-out presentation<br>Head-shelf presentation<br>Basket presentations	In order to build support, store actors were asked to provide input regarding the products selected.
Commercial values	Some store actors desired to use the component trial as a way to develop a stronger public image for their store as a means to draw customers, which they did not feel the HFI contributed enough to	General factor	Discussions were had with these store actors, and regional management, to explain the component designs, the importance of implementing them as planned, and find an acceptable middle ground in their implementation
	Store management perceived certain products selected for presentation components as a commercial risk (e.g., low demand), and often prioritized the presentation of commercially 'more viable' products, or seasonal products, often replacing component presentations with unhealthy products	Check-out presentation<br>Head-shelf presentation<br>Basket presentations	Store managers were allowed to select the products for the basket presentations themselves, from an approved 'healthy' list, although they did not always do so
**Factor**	**Description**	**Affected HFI components**	**Solutions**
	**Organization system practices**		
Friction and Prioritization	Regional and central management showed relatively little interest in the HFI, and apparently pushed stores to prioritize commercially beneficial goals and activities. Furthermore, a lack of direction and involvement was perceived with regards to making the HFI 'work'	General factor	*No feasible solution was found, a message was sent from the central management to participating stores, signaling support of the organization*

	The organization would require stores to place orders for seasonal products a year in advance (before the store for the trial were selected), and as such, these orders had been made without accounting for the space taken up by the presentation components.	Basket presentations	When this problem was noticed, the orders could not be cancelled. Stores were offered additional baskets to expand their presentation spaces, but none took up this offer
	At several points, products targeted by the price mutation component were also part of advertised promotions, leading to discrepancies in advertised prices, and actual prices in participating stores	Price mutation	The researchers would wait until regular promotions were fully set, before finalizing component-related mutations, and communicate with the actors planning regular promotions
Events	Near the end of the component trial, the collaborating supermarket chain started the process for a merger. This led to several participating stores being closed down before the end of the trial, for refurbishing	General factor	*No feasible solution was found*
	The organization was conducting a major reorganization in the hierarchical structures of stores, which included several of the participating stores. This led to various employees receiving new responsibilities and needing to follow training, as well as changes in store management	General factor	*No feasible solution was found*
**Factor**	**Description**	**Affected HFI components**	**Solutions**
**Organization system structures**			
Human Resources	Near the end of the component trial, the collaborating supermarket chain started the process for a merger. This drew away several key figures in the central organization from the project	General factor	Where possible, a replacement was sought by the organization for the tasks of the actor drawn away
Knowledge Resources	The organizational databases did not have a label for which products qualify as ‘healthy,’ and should receive component signing, making it difficult for stores to accurately add shelf tags to new healthy products in the store, and replace those that were damaged or fell down.	Shelf tags	The organizational database was linked with a database supplied by the Netherlands Nutrition Centre, which had indications for which products could be considered ‘healthy’
Processes and systems	The price-management system could not indicate differences between regular price mutations, and those part of the component, making it impossible for store management to distinguish between them (for the purpose of placing component price-cards)	Price cards<br>Price mutations	HFI-related price mutations would be sent on a different day than the regular ones
	The central price-management system suffered from various bugs and problems, leading to price mutations being incorrectly implemented or retracted, and incorrect prices being displayed in stores	Price mutations	A new price-management system was already planned, and its implementation removed the issues caused by the old system
	The implementation of a new price-management system led to several issues with prices in stores during the initial learning period, in which bugs in the system had to be identified and resolved	Price mutations	The price mutations were discontinued for several weeks, until the issues with the system were identified and resolved

Performance metrics	Stores are monitored through various commercial metrics (e.g., losses, wage-costs, hours of labor, turnover), and pushed to improve these (particularly wages and hours). Some components were perceived by store managers to negatively affect such metrics, which may pose a risk to their performance bonusses	General factor	Central office actors promised that store managers' performance bonusses would not be negatively affected by the HFI, and additional work hours would be made available to the stores
**Factor**	**Description**	**Affected HFI components**	**Solutions**
**Organization system culture**			
Commercial values	Stores were under substantial pressure from organizational metrics, programs, and interactions with their superiors to prioritize commercial success, and seemed to view this as their primary goal.	General factor	*No feasible solution was found*
**Factor**	**Description**	**Affected HFI components**	**Solutions**
**External factors**			
Consumer landscape	The healthy products presented and promoted in various components were often in lower demand, and therefore commercially less viable, compared to unhealthier options	Check-out presentation<br>Head-shelf presentation<br>Basket presentations	*No feasible solution was found Outside sphere of influence of involved actors*
External obligations	The organization has various contractual agreements with external parties, such as food manufacturers, which included certain obligations. For example, the presentation of unhealthy products in certain places of the store (e.g., next to the check-out).	Check-out presentation<br>Head-shelf presentation	Alternative places were sought in the stores for these obligatory presentations, but due to space constraints these were not always available
External events	The COVID-19 pandemic led to sickness and forced isolation among store employees, creating frequent employee shortages in stores	General factor	*No feasible solution was found Outside sphere of influence of involved actors*
	Preparations for seasonal holidays added workload for the stores	Head-shelf presentation<br>Basket presentations<br>Shelf cards<br>Price cards<br>Price mutations	
